# What Is the Primary Cause of Individual Differences in Contrast Sensitivity?

**DOI:** 10.1371/journal.pone.0069536

**Published:** 2013-07-26

**Authors:** Daniel H. Baker

**Affiliations:** Department of Psychology, University of York, York, United Kingdom; Lund University, Sweden

## Abstract

One of the primary objectives of early visual processing is the detection of luminance variations, often termed image contrast. Normal observers can differ in this ability by at least a factor of 4, yet this variation is typically overlooked, and has never been convincingly explained. This study uses two techniques to investigate the main source of individual variations in contrast sensitivity. First, a noise masking experiment assessed whether differences were due to the observer’s internal noise, or the efficiency with which they extracted information from the stimulus. Second, contrast discrimination functions from 18 previous studies were compared (pairwise, within studies) using a computational model to determine whether differences were due to internal noise or the low level gain properties of contrast transduction. Taken together, the evidence points to differences in contrast gain as being responsible for the majority of individual variation across the normal population. This result is compared with related findings in attention and amblyopia.

## Introduction

Sensitivity to contrast (variations in luminance within an image) is a fundamental property of the human visual system. The majority of cells in primary visual cortex (V1) respond to contrast at various spatial scales and orientations [1]. At a behavioural level, normal observers are highly sensitive to contrast, being easily able to detect variations (e.g. sinusoidal gratings) of less than 1% of the background luminance under optimal conditions [2]. Research into this ability has been extensive (over 9000 entries in the PubMed database include the term “contrast sensitivity”), and has had a huge impact in the understanding of normal visual processes as well as clinical visual disorders. But despite this interest, nobody has yet attempted to answer a fundamental question: why do some normal observers have greater contrast sensitivity than others?

Individual differences in contrast sensitivity are often overlooked in psychophysical studies, which typically involve few observers (<8) and often average results across observers, obscuring any differences (though see [3], [4] for an alternative approach). However, such differences are apparent when looked for. For example, Schefrin et al. [5] report differences in low spatial frequency scotopic sensitivity of around 0.5 log units (a factor of >3) in healthy young adults, and even greater differences across a wider age range. More recently, Baker & Graf [6] measured the sensitivity of 41 observers for detecting sine-wave gratings at 2c/deg. They found a more than four-fold variation in sensitivity across their population that appeared to correlate with alternation rates in binocular rivalry. Another recent paper [7] reported sensitivity ranges spanning a factor of >20, with interquartile ranges of a factor of ∼2, for two ‘magnocellular’ detection tasks performed on over 1000 subjects. In the present study, data from 18 publications were reanalysed, and again revealed intra-study individual differences approaching a four-fold variation (see below).

There are several possible explanations for the above sensitivity differences. Most obviously, they could be caused by differences in optical blurring, e.g. due to myopia. This seems unlikely for two reasons. First, psychophysical observers typically wear their prescribed optical correction during testing, and so have nominally normal visual acuity. Second, blur has an effect at high spatial frequencies, leaving lower frequencies unaffected (unless it is extreme), yet individual differences are apparent across the entire CSF (e.g. < = 2c/deg in the studies of Schefrin et al. [5] and Baker & Graf [6]). So, blur is not a convincing explanation. Practise effects also seem unlikely, since differences persist between highly experienced observers, and studies on perceptual learning or training indicate that extensive practise provides only a marginal [8] or nonexistent [9]–[12] improvement in sensitivity. Differences in criterion or bias can also be ruled out, since modern studies use bias-free empirical methods (e.g. two-alternative forced choice; note that early work using yes/no or adjustment tasks is vulnerable to criterion effects).

Here, three ‘neural’ limits on visual sensitivity are considered: observer efficiency, internal noise, and gain control nonlinearities. In Part I a noise masking paradigm was used to rule out differences in efficiency. In Part II contrast discrimination functions from 18 studies were reanalysed using a computational model to reveal that individual differences are not primarily due to differences in internal noise. Instead, it appears that contrast gain control nonlinearities are responsible for variations in sensitivity within the normal population.

## Results

### Part I – Noise Masking

Noise masking has been used to understand performance differences in several domains, such as amblyopia [13], peripheral vision [14], attention [15], dyslexia [16] and many others (see [17]). Where a difference in performance exists in the absence of external noise, adding white pixel noise (broadband ‘TV snow’) can allow attribution of this difference to a variation in either internal noise, or the efficiency with which information is extracted from the stimulus [18].

**Figure 1:**
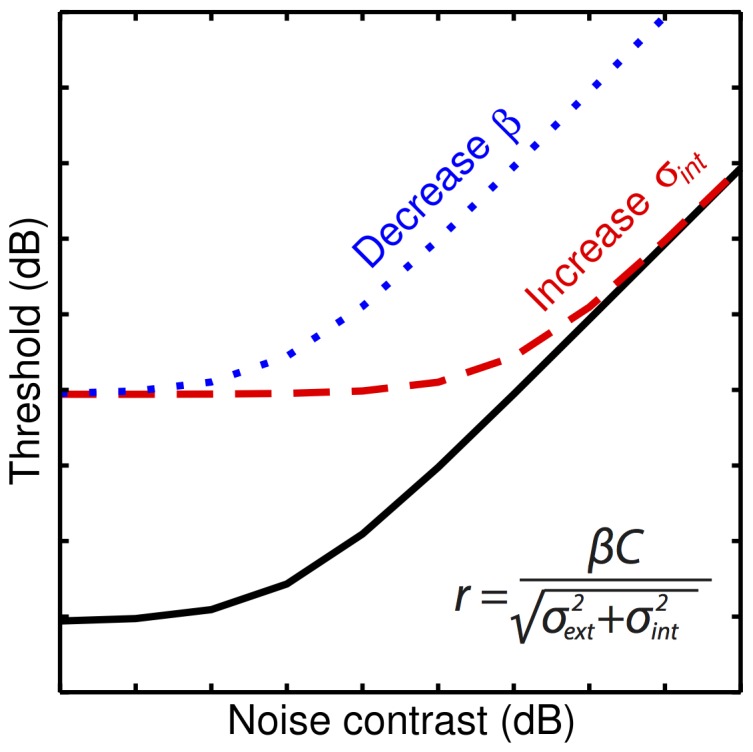
Canonical noise masking functions showing the effect of changing model parameters. The dashed and dotted curves show the effect of changing the level of internal noise (*σ_int_* in equation 2– see Materials and Methods section) or the observer’s efficiency (*β* in equation 2), relative to the solid curve.

The noise masking paradigm assumes a noisy linear observer model, in which the filter (e.g. neural) response to the stimulus is corrupted by additive internal noise. Under this model, variations in internal noise will produce (i) sensitivity differences in the absence of external noise, and (ii) equal sensitivity when external noise is strong. In other words, noise masking functions will converge at high noise levels (compare solid and dashed curves in Figure 1). Alternatively, differences in the efficiency with which information is extracted from the stimulus (e.g. the match of the perceptual template) will produce vertical translations of the noise masking function (compare solid and dotted curves in Figure 1).

This technique was applied using four varieties of external noise, for two observers with a substantial, stable difference in sensitivity. Four varieties of noise were used for several reasons. There is evidence that pixel noise also produces suppression via contrast gain control [19], so a 0D noise condition (see Methods) that avoids this was also included. However, it seemed worthwhile to also include more standard noise masks for comparison with previous studies. Finally, showing the same behaviour consistently across diverse mask types makes the findings more convincing.

**Figure 2:**
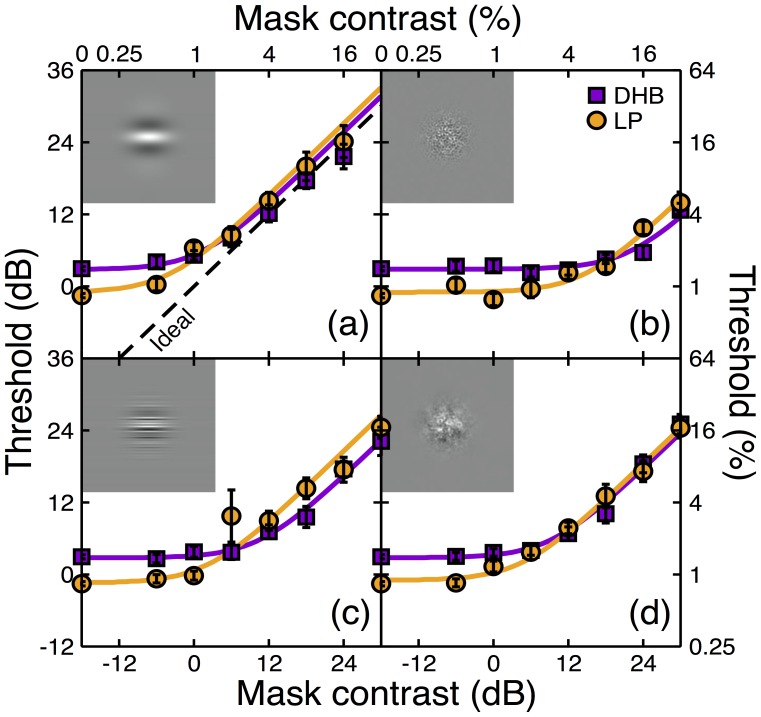
Noise masking functions for two observers and four varieties of external noise. Insets to each panel show examples of the noise stimuli. (a) 0D noise, (b) 2D white noise, (c) 1D white noise, (d) 2D pink noise. The 0D noise had the same spatial waveform as the target. Error bars show standard deviations of a population of bootstrap resamples. The curves are fits of a noisy linear observer model detailed in the text, that had two free parameters per curve. The oblique dashed line in panel (a) gives the prediction of the ideal observer.

The results for all four mask types are shown in Figure 2, with curves giving two-parameter fits of equation 1. The inter-observer difference in sensitivity at threshold (noise contrast of 0%) of around a factor of 2 is clear, and remains stable at the lower mask contrasts in each panel. At higher mask contrasts the masking functions approximately converge, consistent with a difference in internal noise (see Figure 1). Masking functions have a slope of unity, consistent with previous results [17] and theoretical expectations [18], [20]. The poorer performance for observer DHB (purple squares) at threshold does not persist at high mask contrasts. Indeed, for some mask types (0D noise and 1D white noise), observer DHB appears to be slightly more sensitive at high mask levels than observer LP (orange circles). This is consistent with a small difference in the efficiency parameter (*β* is slightly larger for DHB, see Table 1), but it is clear that the main cause of poorer threshold performance for DHB is the internal noise parameter (*σ_int_*), that is on average twice as large for DHB relative to LP (see Table 1).

**Table 1:** Parameters for best fits of equation 2 to the data in Figure 2, and ratios of those parameters across observers (bold).

	0D noise	2D whitenoise	1D whitenoise	2D pinknoise	Mean
*β* (DHB)	1.07±0.36	8.37±1.07	2.34±0.33	2.00±0.19	
*β* (LP)	0.67±0.15	5.68±0.93	1.54±0.24	1.79±0.21	
***β*** ** ratio**	**1.59**	**1.47**	**1.52**	**1.12**	***1.43***
*σ_int_* (DHB)	1.71±0.36	12.21±1.70	3.43±0.58	2.90±0.33	
*σ_int_* (LP)	0.60±0.27	5.35±1.16	1.48±0.32	1.69±0.27	
***σ_int_*** ** ratio**	**2.85**	**2.28**	**2.32**	**1.72**	***2.29***

Standard deviations of the bootstrapped parameter values are also given based refitting the model to synthetic data sets generated by 1000 bootstrap resamples per empirical threshold.

Comparison of noise masking functions for two observers, using four varieties of external noise, implies that the main source of variability is in the level of internal noise. This makes a major role for differences in calculation efficiency unlikely, at least between these two observers. But to what extent does the conclusion of internal noise differences rely on the assumption of a noisy *linear* observer? The following section demonstrates that well established nonlinearities of early visual processing suggest two equally plausible interpretations of apparent differences in the *σ_int_* parameter.

### Beyond a Linear Observer

There is abundant evidence that the human response to contrast is not linear, but instead accelerates at low contrasts, and saturates at high contrasts. This nonlinearity is consistent with contrast discrimination data (e.g. [21]), fMRI responses [22] and ERP recordings [23]. It most likely emerges from the combined output of many single neurons, with properties similar to those found in visual cortex [24], [25].

**Figure 3:**
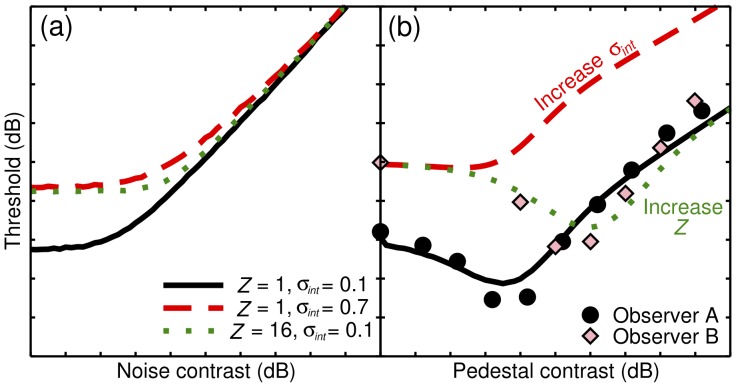
Behaviour of a nonlinear gain control model for noise masking (a) and contrast discrimination (b). The data in (b) are replotted from Henning & Wichmann [62] and are for observers NAL (circles) and GBH (diamonds). In both panels, green dotted curves show the effect of increasing the *Z* parameter of equation 1, and red dashed curves show the effect of increasing *σ_int_*, relative to the solid curves.

A widely used equation [21] that describes the contrast response function is:
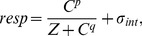
(1)where *C* is target contrast, the exponents *p* and *q* have values of 2.4 and 2 respectively, *Z* is a constant (often termed the saturation constant), and *σ_int_* is the observer’s internal noise (see above). This equation can be used stochastically to simulate performance in noise masking experiments by making *C* equal to the activity in the detecting mechanism (e.g. target plus mask), and sampling *σ_int_* from a zero-mean normal distribution, on each interval of every trial (see [20]). However, doing so reveals a problem of interpretation for noise masking experiments. In Figure 3a, comparing the solid curve to the dotted curve shows the effect of increasing the saturation constant, *Z*, whereas the dashed curve shows the effect of increasing the level of internal noise, *σ_int_*. It is clear that both of these parameters shift the noise masking function upwards and to the right, such that the handles of the masking functions converge. This means that empirical results such as those in Figure 2 could be produced by a change in either parameter. Estimates of *σ_int_* from noise masking experiments are therefore relative, not absolute, and are confounded by differences in *Z*.

### Part II – Contrast Discrimination

Although both the saturation and internal noise parameters in equation 1 affect the signal-to-noise ratio of the model (one by reducing signal, the other by increasing noise), their operations are distinct: *Z* changes the gain of the nonlinearity at low contrasts, whereas *σ_int_* determines the increase in activity required to reach threshold. Noise masking experiments cannot distinguish between these two possibilities (Figure 3a), but an alternative paradigm exists that can.

Contrast discrimination experiments, in which observers detect a contrast increment on a fixed contrast ‘pedestal’, tap directly into the contrast response function (they measure its derivative). The characteristic ‘dipper’-shaped nonmonotonic functions produced by such experiments [26], [27] are affected in different ways by the two parameters. Increasing *Z* translates the dipper upwards and to the right, resulting in converging dipper handles (compare solid and dotted curves in Figure 3b), whereas a change in *σ_int_* shifts the dipper vertically (compare solid and dashed curves in Figure 3b).

A clear advantage of using contrast discrimination functions to investigate individual differences is that many existing studies contain such data for a number of observers. A non-exhaustive search produced 18 studies, and 63 dipper functions in total (see Figure S1). By fitting equation 1 to these data, it is possible to estimate whether individual differences in detection threshold are mainly due to differences in either *Z* or *σ_int_*. This was done by first fitting the equation to the entire dipper function of one observer, with both parameters free (see circles and solid curve in Figure 3b; the exponents *p* and *q* were fixed at standard values of 2.4 and 2). Each parameter was then adjusted separately to exactly predict only the detection threshold of a second observer (leftmost pink diamond in Figure 3b). These two versions of the model, one where *Z* was changed (dotted curve), one where *σ_int_* was changed (dashed curve), then predict performance over the remainder of the dipper function for the second observer with no further degrees of freedom. By comparing the accuracy of these two predictions (assessed by the RMS error, see Materials and Methods), the parameter that best explains individual differences at detection threshold is revealed (in Figure 3b it is clearly a change in *Z*).

**Figure 4:**
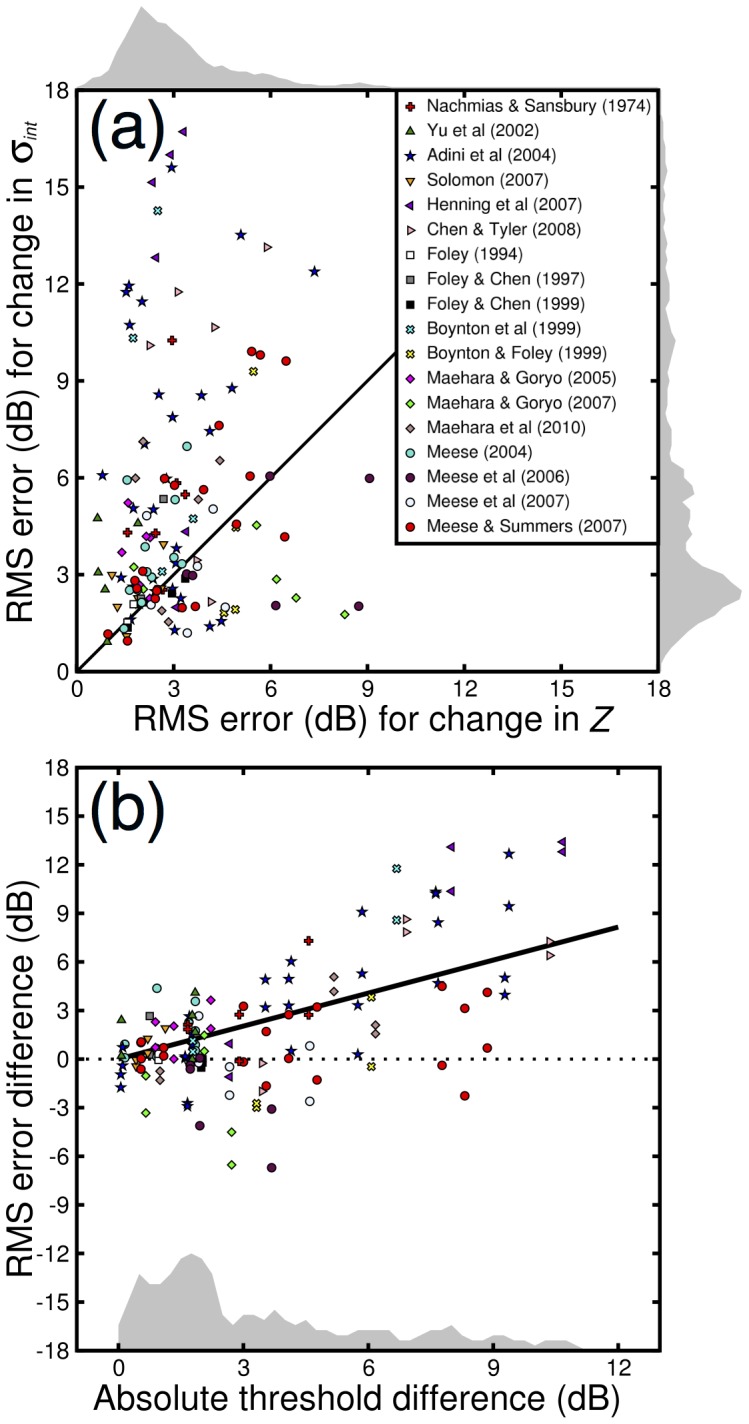
Scatterplots showing RMS errors for fits to 138 pairs of dipper functions. In (a), points above the oblique line indicate that changing *σ_int_* produced a worse fit (larger error) than changing *Z*. In (b), the difference between the two errors is plotted against the absolute difference in detection threshold for each pair of dippers. Points above the dotted line correspond to points above the oblique line in panel (a). For pairs with the largest threshold differences (e.g. >6dB) almost all points favour the change in *Z*. The solid black line is the best fitting regression line, constrained to pass through [0,0], and has a slope of 0.68. The shaded histograms in each panel show the density of points. Since these exhibit positive skew, the data were log-transformed before performing statistical tests.

Figure 4a shows a scatterplot of RMS errors indicating the relative success of manipulating the two parameters. Points falling below the diagonal line indicate that differences in internal noise (*σ_int_*) best explain individual variation in detection thresholds, whereas points above the line support differences in the gain parameter, *Z*. It is clear that the majority of points (95/138) fall above the line, often markedly so. This difference is highly significant (paired t-test on log values, *t_137_* = 5.6, *p*<0.01).

It is also interesting to ask whether the greater predictive power of *Z* is related to the magnitude of the threshold difference to be explained. This can be assessed by plotting the error difference against the absolute difference in detection threshold for each pair of observers (Figure 4b), it is clear that the largest differences (e.g. those >6dB) are almost all best described by a change in *Z*. Performing linear regression on the data in Figure 4b with the regression line constrained to pass through [0,0] revealed the significant positive relationship (*R^2^* = 0.62, *p*<0.001) shown by the solid black line. The significantly positive slope confirms the hypothesis that changing the *Z* parameter provides the better description of the data.

Fitting 63 dipper functions using a computational model, and making pairwise comparisons between them, demonstrates that the major source of individual differences at detection threshold is the saturation constant, *Z*. Although there will inevitably be contributions from other factors, such as internal noise, optical blur, template efficiency and attention, the principal individual difference appears to be in the low level gain properties of contrast transduction.

**Figure 5:**
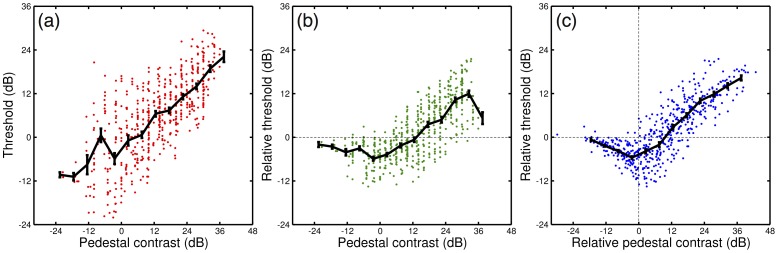
Summary of 63 dipper functions, plotted three ways. Panel (a) shows the raw data from 18 studies (dots) and a binned average (black line, bin width of 6dB). Panel (b) shows the same data with the thresholds (y-axis) normalized to the baseline detection threshold (i.e. pedestal contrast of 0%) for each observer. Panel (c) shows the same data but with both axes normalized to the baseline detection threshold. Error bars show ±1SE of the mean for each bin.

An alternative analysis of the contrast discrimination data leads to the same conclusion. Figure 5a shows all of the raw (un-normalized) contrast discrimination data (i.e. pedestal contrasts >0%, so omitting baseline detection thresholds) plotted in a single panel. If the main source of differences were due to internal noise (*σ_int_*), the vertical translation that this implies (compare solid and dashed curves in Figure 3b) could be compensated for by normalizing thresholds (the y-axis) by the baseline detection threshold for each observer. This is shown in Figure 5b, and on average (solid line) does produce a better approximation of a dipper function than the raw data (Figure 5a).

It is much less convincing than the average function in Figure 5c, however. This was produced by normalizing both thresholds and pedestal contrasts to the baseline detection thresholds, so that both axes are in relative units [28]. This is approximately consistent with the diagonal translation of the dipper produced by varying the saturation parameter (*Z*) (note that because the slope of the dipper handle is typically <1, these two operations are not precisely identical, but they are sufficiently similar for the present exposition). The average function has several familiar properties. The slope of the ‘handle’ region (calculated by linear regression) is 0.6, consistent with previous work [21]. Furthermore, the lowest point of the facilitatory ‘dip’ region occurs at around the detection threshold for the target alone (0dB on these normalized axes). These observations support the main conclusions from the previous section.

## Discussion

The main source of individual differences in contrast sensitivity was investigated using noise masking experiments, and by re-analysis of a large corpus of contrast discrimination data. Neither observer efficiency or internal noise appear to be primary factors. Instead, the evidence indicates that gain properties of the system are responsible for individual variation. The following section considers sensitivity differences in three other situations, discusses possible causes of individual differences, and alternative interpretations of the present findings.

### Attention

Using a similar logic to the comparison of dipper functions above, Huang & Dobkins [29] measured contrast discrimination under conditions of attention and inattention. Their most striking finding was that inattention (induced by observers performing a resource intensive concurrent task) caused a vertical shift of the dipper function, consistent with a change in internal noise (see also [30] for similar findings and [31] for a counterexample).

The finding that attention modulates internal noise rather than contrast gain means that individual differences in attention (or motivation) are unlikely to explain the present dipper results. Interestingly, Huang and Dobkins [29] attribute their findings to a change in response gain (e.g. a multiplicative scaling of the output from the nonlinear transducer), which is mathematically indistinguishable from a change in internal noise in the dipper paradigm. However, the noise interpretation is supported by other work (e.g. [32]) which has demonstrated that attention appears to primarily reduce internal noise (or, equivalently, to decorrelate the spontaneous firing of neurons [33], [34]), rather than amplify internal responses.

### Adaptation

When a stimulus is viewed for a long period of time, sensitivity to subsequent similar stimuli is reduced [35]. Several studies have shown that this adaptation effect corresponds to a change in contrast gain (the *Z* parameter), producing a diagonal shift in the dipper function [36]–[39]. Since the visual system continually adapts to its surroundings (e.g. [40]), it may be that individual differences are influenced by some global adaptation state, perhaps determined during development. Alternatively, adaptation might operate over shorter timescales, and vary across observers. For low spatial frequency stimuli, adaptation to (or masking from [41]) the mean luminance of the display is a potential candidate mechanism to explain individual differences.

### Amblyopia

In amblyopia, contrast sensitivity can be severely impaired in one eye, but normal in the other [42]. Several studies have attempted to account for this deficit using the noise masking paradigm, with mixed results. For example, Huang, Tao, Zhou and Lu [43] concluded that some amblyopes differed in efficiency, whereas others differed in internal noise. Pelli, Levi and Chung [44] found weak evidence for increased internal noise, but attribute most of the deficit for letter identification to reduced efficiency, whereas other work concludes that greater internal noise is responsible [45], [46].

The above insights regarding the usefulness of dipper functions could be informative here: based on available data, is it possible that apparent differences in internal noise are in fact due to changes in contrast gain? Two studies have compared monocular contrast discrimination functions in both eyes of amblyopes [47], [48]. In both studies, there is clear evidence of a vertical translation of the dipper functions, such that the handles do not converge at high contrasts (see e.g. Figure 3i of [47] and Figure 6 of [48]). This is consistent with greater internal noise in the amblyopic eye only, a conclusion also reached by Baker et al. [47] using computational modelling.

**Figure 6:**
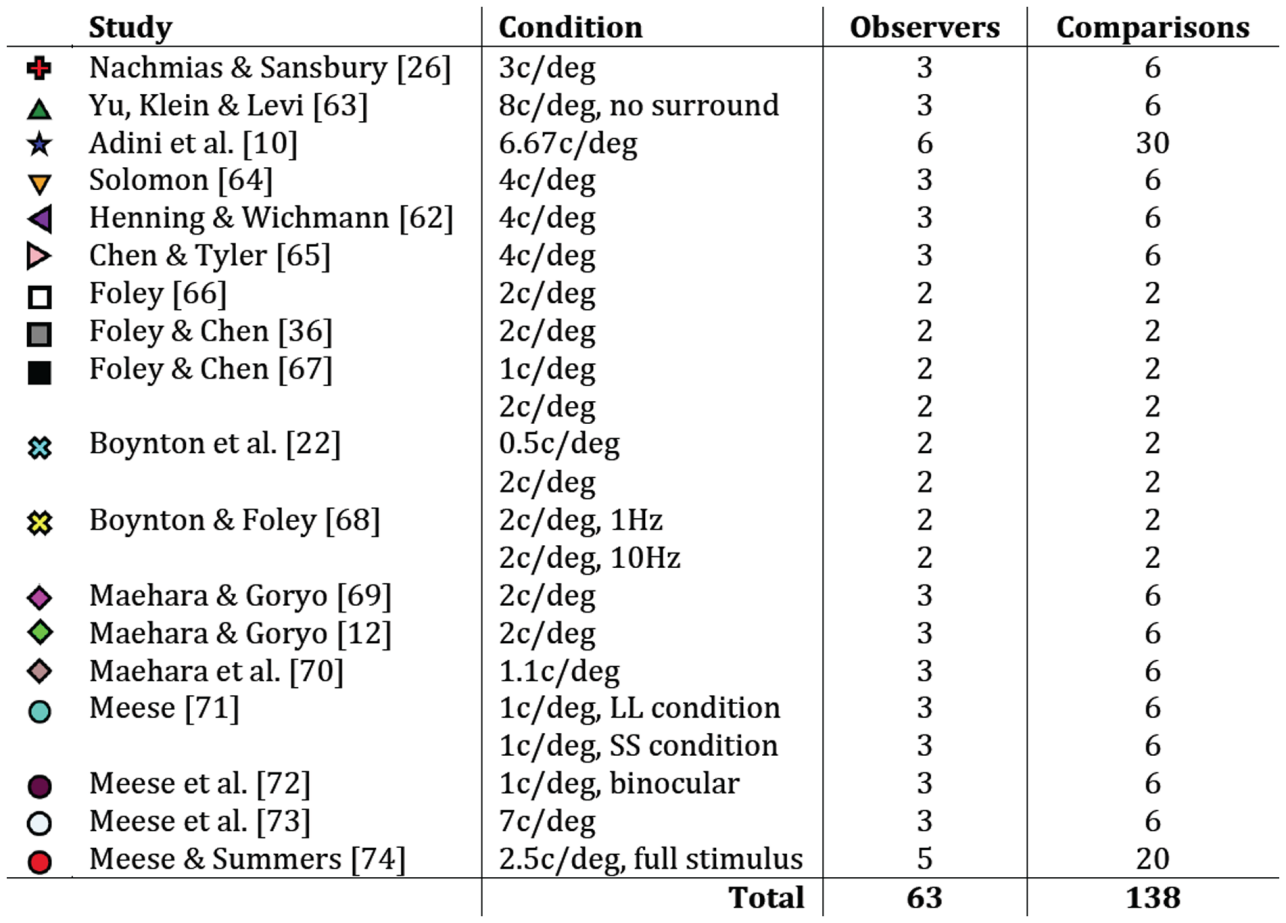
Details of 18 studies that contained dipper functions used in the meta-analysis. The number of pairwise comparisons is determined by n*(n-1), where *n* is the number of observers who completed a given condition.

### Source of Differences

It is reasonable to ask what might be responsible for the differences in contrast gain between individuals. It is well established that contrast sensitivity follows a developmental trajectory [49], improving until around the age of 12 [50]. (Note that the decline in contrast sensitivity in later life is typically confined to high spatial frequencies (e.g. [2], [51]) and so is most likely optical in origin). Conceivably, environmental differences during development might affect adult sensitivity, perhaps owing to prolonged adaptation to a restricted range of contrasts [40]. As an extreme example, kittens reared in impoverished visual environments containing stripes of only one orientation exhibit long lasting behavioural and morphological visual abnormalities [52]. Alternatively, there could be genetic factors that determine contrast sensitivity, perhaps mediated by neurotransmitter levels, which have recently been shown to predict individual differences in bistable perception [53]. Though this area is in its infancy, there is at least one major study on the genetics of perception currently underway that may shed light on such issues (see [54]).

A series of studies by Peterzell and colleagues exploited individual differences in sensitivity to infer the structure of psychophysical channels, using a factor analysis technique [3], [4]. The assumption behind this method is that individual channels will vary in their sensitivity between observers, but that such differences will be largely uncorrelated within an observer. Such an assumption is consistent with differences in gain or channel-specific noise, but not with differences in global decision noise or uncertainty (see next section).

A very different conclusion was reached by Halpern, Andrews & Purves [55]. These authors measured performance for a group of 20 observers on a battery of visual tasks. These included orientation, wavelength and motion direction judgements, as well as contrast discrimination (increment detection). They performed principal components analysis on the results, and identified a single factor that correlated highly with visual performance on all tasks apart from motion direction detection and Snellen acuity. Within the present context, an index of general visual ability maps more naturally onto differences in uncertainty, decision noise or even general intelligence. This is clearly distinct from the major source of differences in contrast detection thresholds identified in the present study.

### Contrast Gain or Uncertainty?

In this study, contrast discrimination has been explained in terms of nonlinear contrast gain control. However, there is an alternative explanation for dipper functions that posits a linear observer who is uncertain about the precise properties of the signal [56], and so monitors both relevant and irrelevant (noisy) mechanisms. In this model, facilitation (the dip) occurs because the pedestal increases the activity of the relevant mechanisms above the noise of the irrelevant mechanisms, and so reduces uncertainty. Pedestal masking (the dipper handle) is typically attributed to a separate process of multiplicative (i.e. signal-dependent) noise [57], [58].

Under this framework, differences in contrast gain are reinterpreted as differences in intrinsic uncertainty between observers. This is difficult to rule out categorically, though it should be noted that (i) the uncertainty/multiplicative noise model would still reject additive internal noise as an explanation for individual differences, and (ii) highly ‘certain’ (and thus apparently more sensitive) observers should show little or no facilitation (i.e. a shallow dip), yet there appears to be no evidence of this (e.g. the two observers in Figure 3b have similar sized dips). Furthermore, explaining the sometimes large individual differences in sensitivity (up to a factor of 4, see Figure 4b) entirely in this way implies variations in uncertainty of approximately a factor of 1,000,000 (see Figure 9 of [59]). This seems highly improbable.

### Conclusions

Understanding individual differences in performance is important for a number of high-precision task domains, as well as being of interest in basic and clinical research. This study has investigated, for the first time, the main source of individual differences in contrast sensitivity. Differences in internal noise and efficiency are ruled out. Instead, observers appear to differ in the low level gain parameters of the visual system.

## Materials and Methods

### Ethics Statement

All participants gave written informed consent, and procedures were approved by the Aston University Ethics Committee.

### Apparatus & Stimuli (part I)

All stimuli were presented on an Iiyama VisionMaster Pro 510 running at 85 Hz. A BITS++ box (Cambridge Research Systems Ltd., Kent, UK) was used to provide 14-bit greyscale resolution. The monitor was gamma-corrected using a photometer and had a mean luminance of 50cd/m^2^. At the viewing distance of 114 cm, one degree of visual angle subtended 60 monitor pixels.

The target stimulus was a horizontal 1c/deg log-Gabor patch in positive cosine phase, with bandwidths of ±25° and 1.3 octaves (see [60]). There were three varieties of pixel noise mask: 1D and 2D white noise, and 2D pink noise. Each began as a 2D array of zero-mean Gaussian noise. All were low-pass filtered in the Fourier domain (high frequency cut-off of 15c/deg), and for the pink noise the slope of the amplitude spectrum was adjusted to *1/f*. They were then inverse-transformed, and windowed in the spatial domain using a 2D Gaussian window that had the same spatial extent as the target (full-width-at-half-height of 1°).

The fourth type of noise, termed 0D noise, was a pedestal of random contrast, determined on an interval-by-interval basis from a zero-mean Gaussian distribution (where negative values constitute a phase reversal). It was therefore spatially identical to the target (i.e. the noise energy was distributed across 0 spatial dimensions), but of variable contrast. See [19] for further details of this condition. Example noise stimuli are shown in the insets to Figure 2.

Contrast is reported in dB units, where *C_dB_  = 20log_10_(C_%_).* For the target stimulus, *C_%_* is the delta (or Weber) contrast of the stimulus (*ΔL/L_0_*). For the pixel noise stimuli, *C_%_* is the RMS contrast of the mask (equivalent to the luminance standard deviation), expressed as a percentage. For the 0D noise, *C_%_* is the standard deviation of the Gaussian noise source that determined the interval-by-interval mask contrasts.

### Procedure

A two-interval-forced-choice (2IFC) paradigm was used, with target contrast controlled by a pair of 3-down-1-up staircases. Stimuli were presented in the centre of the monitor for 100 ms, with an interstimulus interval of 400 ms. In one interval a noise mask was presented alone, and in the other interval a different sample of noise was presented combined with the target stimulus. The observer’s task was to identify, using a two-button mouse, which interval contained the target. Each interval was marked by a beep, but no feedback was given to indicate response accuracy. A quad of fixation points was visible throughout to indicate the stimulus location.

Seven mask contrasts were used for each mask type, and detection thresholds (with no mask) were also measured. Each observer repeated the experiment four times, and thresholds (estimated by fitting cumulative Gaussian functions to the staircase data) were averaged across repetitions.

The masking functions for the 0D noise and 2D white noise conditions have previously been reported [19]. In that study, they were used for a very different purpose (to estimate equally effective mask contrasts across the two conditions for use in subsequent experiments), so this is the first direct comparison across observers. The additional conditions (1D white and 2D pink noise) have not previously been reported.

### Observers

Two experienced psychophysical observers completed all noise masking conditions. Both were emmetropic, with normal binocular vision, and aged 29 at the time of testing. They were selected because they differ in foveal contrast sensitivity by around a factor of 2 at low spatial frequencies (1c/deg), as observed in previous work (Figures 4, 5 and A1 in [61]). This difference has remained stable over the past five years.

### Model Fitting (part I)

The results were fitted by a widely used deterministic approximation of a noisy linear observer model (e.g. [17]; see also [20] for further discussion). The model response is given by,
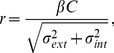
(2)where *C* is target contrast, *σ_ext_* is external noise variance, and *σ_int_* and *β* are free parameters representing the observer’s internal noise variance and calculation efficiency, respectively. Threshold is reached when the response, *r*, is greater in the target interval than in the null interval by some fixed quantity, here implicitly unity. The model was fitted with two free parameters per function by minimising the root-mean-squared (RMS) error between model and data. The RMS error in decibels (dB) is defined as 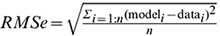, where *n* is the number of data points. Smaller errors indicate better fits.

### Details of Computational Analysis (part II)

Data were obtained from 18 published studies that either plotted results for individual observers separately, or were carried out in the Aston vision laboratory (meaning that the individual observer data were available even if they were not presented in the published paper). In many cases, data were estimated from published figures using a computer program (see Figure S1). All studies measured contrast discrimination functions using standard forced-choice methods at a range of spatial frequencies (see Figure 6, and the individual studies for details). The fitting procedure described in the Results section was repeated for pairwise combinations of observers within a given experiment, ensuring that all methodological details were constant (138 comparisons). As previously, model predictions were compared by calculating the RMS error for each fit.

Note that for the contrast discrimination predictions an analytic version of equation 1 was used. Deterministic responses were calculated for the pedestal only interval, and the pedestal+target interval across a range of target contrasts. Threshold was defined as the target contrast that increased the model response by a fixed amount, which was proportional to *σ_int_*. This is exactly equivalent to the predictions of a very large number of stochastic simulations, but is sufficiently computationally tractable to permit parameter optimization.

## Supporting Information

Figure S1 **Dipper functions from 18 studies.** Curves show the best fit of a gain control model with two free parameters. The mean RMS error of the fits was 1.53dB. These initial fits were then adjusted to predict the results for the other observers in each study, as detailed in the body of the manuscript. Details of the conditions for each study are given in Figure 6 and the methods sections of the source publications.
(TIFF)
