# Supplementary figures and images for: What Is the Primary Cause of Individual Differences in Contrast Sensitivity?
Source: PLoS One. 2013 Jul 26;8(7):e69536. doi: 10.1371/journal.pone.0069536 (PMC3724920; doi:10.1371/journal.pone.0069536)

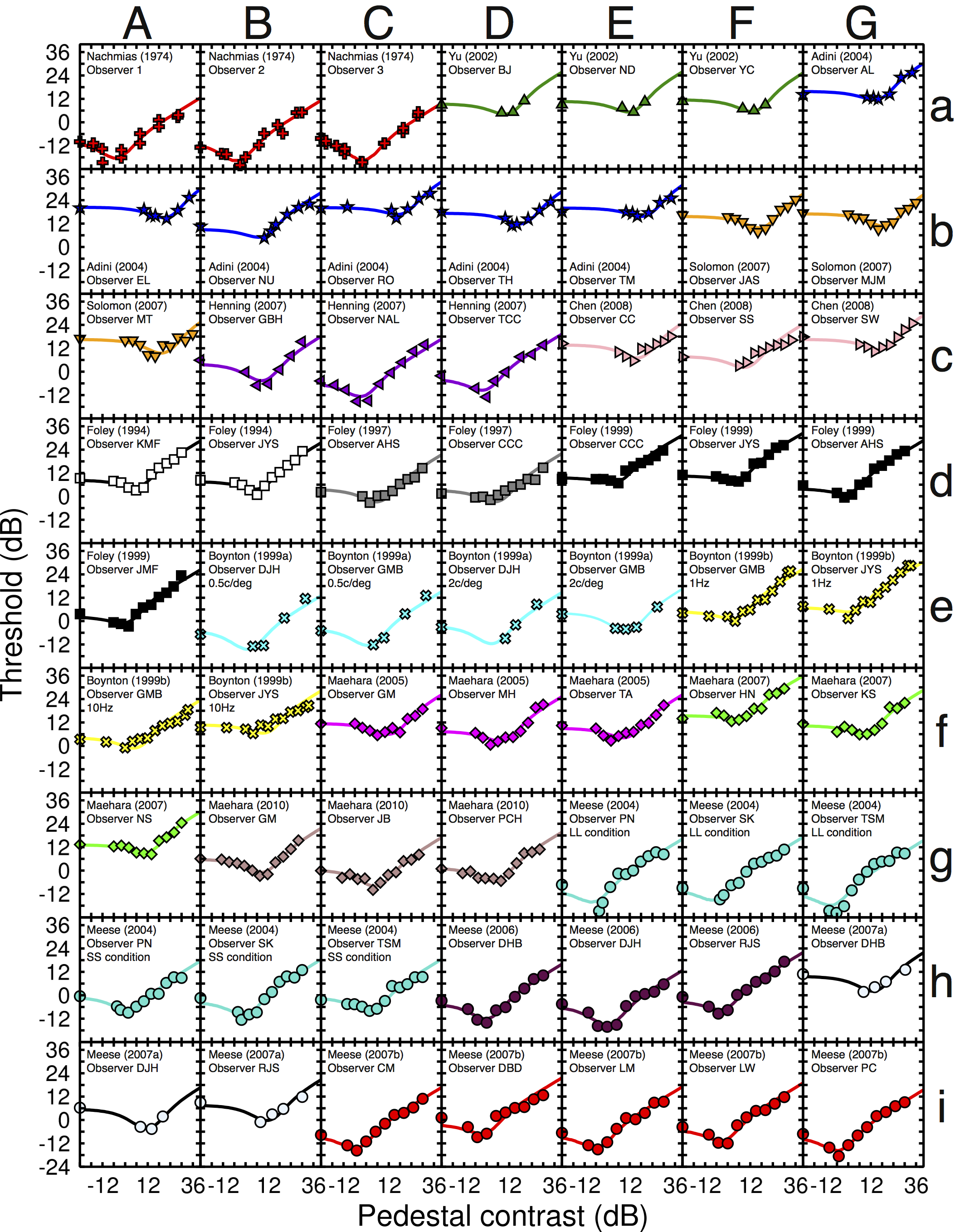

Supplement: Figure S1 — Dipper functions from 18 studies. Curves show the best fit of a gain control model with two free parameters. The mean RMS error of the fits was 1.53dB. These initial fits were then adjusted to predict the results for the other observers in each study, as detailed in the body of the manuscript. Details of the conditions for each study are given in Figure 6 and the methods sections of the source publications. (TIFF) [file pone.0069536.s001.tiff]
